# Exuberant Dermatitis Herpetiformis With Oral Involvement Mimicking Behçet Disease and Disseminated Herpetic Infection: The Crucial Role of Direct Immunofluorescence

**DOI:** 10.7759/cureus.112573

**Published:** 2026-07-13

**Authors:** Carla F Genevcius, Daniel A Santos

**Affiliations:** 1 Dermatology, Fundação Educacional do Município de Assis, Assis, BRA; 2 Medicine, Fundação Educacional do Município de Assis, Assis, BRA

**Keywords:** autoimmune blistering disease, dermatitis herpetiformis, diagnostic challenge, direct immunofluorescence, gluten-sensitive enteropathy

## Abstract

Dermatitis herpetiformis (DH) is a chronic autoimmune blistering dermatosis associated with gluten-sensitive enteropathy and characterized by intensely pruritic papulovesicular eruptions, typically distributed symmetrically on extensor surfaces. Although DH classically presents with grouped vesicles and excoriated papules, atypical and exuberant manifestations may represent a substantial diagnostic challenge, frequently mimicking infectious, inflammatory, and autoimmune blistering disorders.

We report the case of a 30-year-old woman presenting with a one-year history of progressive, intensely pruritic vesiculobullous and erosive lesions involving the trunk and extremities, accompanied by painful oral ulcerations, hemorrhagic crusts, extensive excoriations, secondary bacterial infection, and diffuse post-inflammatory hyperpigmentation. The patient was initially diagnosed with Behçet disease and subsequently hospitalized for suspected disseminated herpetic infection, receiving systemic corticosteroids and intravenous acyclovir without significant clinical improvement.

Dermatological examination revealed widespread erosive and vesiculobullous lesions associated with severe inflammatory activity and oral aphthoid ulcerations. Histopathological examination of active lesions demonstrated intraepidermal blister formation associated with eosinophilic infiltrate and scattered acantholytic cells, findings that initially suggested alternative autoimmune blistering disorders. Direct immunofluorescence (DIF) demonstrated characteristic granular IgA deposition predominantly localized at the tips of dermal papillae, confirming the diagnosis of DH despite atypical histopathological findings. Serological evaluation further revealed positive anti-tissue transglutaminase and anti-endomysial antibodies, corroborating the diagnosis.

Treatment with dapsone combined with a strict gluten-free diet resulted in marked clinical improvement. This case highlights the importance of clinicopathological and immunopathological correlation in atypical presentations of autoimmune blistering diseases and underscores the essential role of DIF in establishing the diagnosis of DH.

## Introduction

Dermatitis herpetiformis (DH), also known as Duhring-Brocq disease, is a chronic autoimmune blistering disorder strongly associated with gluten-sensitive enteropathy and celiac disease [1]. It represents the specific cutaneous manifestation of celiac disease and results from granular IgA deposition at the tips of dermal papillae [1].

Clinically, DH is characterized by intensely pruritic grouped papules and vesicles symmetrically distributed on extensor surfaces, particularly the elbows, knees, buttocks, scalp, and lumbosacral region [2]. Because of severe pruritus and chronic excoriation, intact vesicles are often absent at presentation, making diagnosis challenging [2,3].

Diagnosis relies on clinicopathological correlation and direct immunofluorescence (DIF), which classically demonstrates granular IgA deposition within dermal papillae and is considered the diagnostic gold standard [4]. Histopathological examination typically reveals subepidermal blister formation associated with neutrophilic microabscesses within dermal papillae [5]. Nevertheless, atypical histopathological findings may occur in excoriated, manipulated, or secondarily infected lesions [5].

Exuberant and atypical forms of DH are uncommon and may mimic autoimmune blistering diseases, inflammatory disorders, and disseminated infections, resulting in delayed diagnosis and inappropriate therapeutic approaches [2,6]. Oral involvement is distinctly uncommon and may further complicate diagnosis because of overlap with disorders such as Behçet disease, pemphigus vulgaris, linear IgA bullous dermatosis, and disseminated viral infections [7]. Only isolated cases of oral involvement in DH have been described in the literature, further emphasizing the rarity and diagnostic challenge of this presentation [7]. The present case is remarkable because of the coexistence of extensive erosive lesions, painful oral ulcerations, severe inflammatory activity, secondary bacterial infection, and atypical histopathological findings. These clinical and histopathological characteristics strongly favored alternative diagnoses, including Behçet disease, pemphigus vulgaris, and disseminated herpetic infection.

Despite being considered the specific cutaneous manifestation of celiac disease, DH may occur in the absence of overt gastrointestinal symptoms, further contributing to diagnostic delay. Therefore, recognition of atypical clinical presentations is essential to avoid inappropriate investigations and therapeutic interventions.

We report an exuberant presentation of DH with oral involvement that was initially misdiagnosed as Behçet disease and subsequently treated as disseminated herpetic infection, emphasizing the crucial role of DIF in establishing the correct diagnosis.

## Case presentation

A 30-year-old woman from Marília, São Paulo, Brazil, presented with a one-year history of progressive pruritic blistering skin lesions associated with painful oral ulcerations.

The disease initially manifested as intensely pruritic erythematous papules and vesicles involving the trunk and extremities, progressively evolving into painful erosions, crusted lesions, ulcerated plaques, and extensive residual post-inflammatory hyperpigmentation. The patient reported severe pruritus, a burning sensation, sleep impairment, and a marked deterioration in quality of life. She denied fever, gastrointestinal symptoms, weight loss, arthralgia, genital ulcers, ocular symptoms, or previous autoimmune diseases. No ocular manifestations suggestive of Behçet disease were identified during evaluation.

Before dermatological evaluation, the patient had been followed by another medical specialty and received a presumptive diagnosis of Behçet disease because of the coexistence of oral ulcerations and extensive cutaneous lesions. Systemic corticosteroids were prescribed without satisfactory clinical response. Because of persistent disease activity and therapeutic failure, the patient was referred for dermatological evaluation.

As the disease progressed, extensive erosive and crusted lesions with signs of secondary bacterial infection developed, raising concern for disseminated viral infection. The patient was subsequently hospitalized at another institution and treated with intravenous acyclovir for presumed disseminated herpetic infection, again without significant clinical improvement.

**Figure 1:**
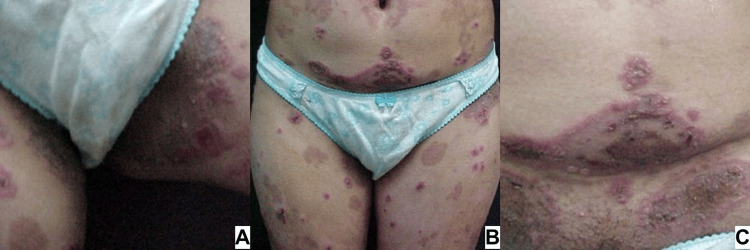
Extensive cutaneous involvement at presentation. (A) Erosive and crusted lesions involving the inframammary region. (B) Multiple excoriated papules, erosions, and post-inflammatory hyperpigmented macules affecting the lower abdomen, groin, and thighs. (C) Detail of grouped crusted and erosive papules on the trunk.

**Figure 2:**
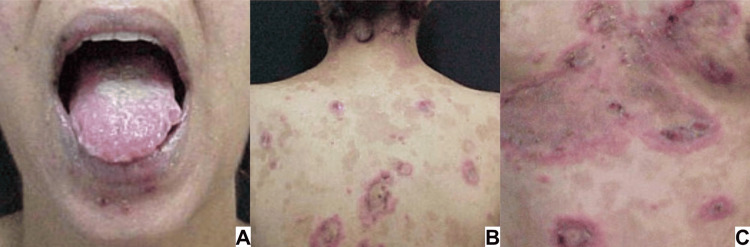
Mucosal and widespread cutaneous involvement. (A) Painful aphthoid ulceration involving the oral mucosa. (B) Widespread excoriated papules, erosions, and crusted lesions involving the posterior trunk. (C) Erosive and crusted lesions on the trunk.

Dermatological examination revealed extensive polymorphic cutaneous involvement, including erythematous papules, vesicles, crusted erosions, and excoriated lesions distributed over the trunk and extremities, as shown in Figures 1A-1C. DIF demonstrated granular IgA deposition along the dermal papillae, supporting the diagnosis of DH (Figure 2A). Extensive excoriations, hemorrhagic crusts, erosions, and signs of secondary bacterial infection were present (Figures 1A, 1C, 2B, 2C). Multiple residual hyperpigmented macules measuring approximately 1 to 10 cm in diameter were distributed throughout the affected areas. The palms and soles were spared. Oral examination demonstrated painful aphthoid ulcerations involving the oral mucosa (Figure 2A).

Laboratory evaluation demonstrated mild inflammatory activity without significant systemic abnormalities. No gastrointestinal manifestations suggestive of celiac disease were reported. Serological investigation for celiac disease revealed positive anti-tissue transglutaminase and anti-endomysial antibodies, further supporting the diagnosis of DH and its association with gluten-sensitive enteropathy.

**Figure 3:**
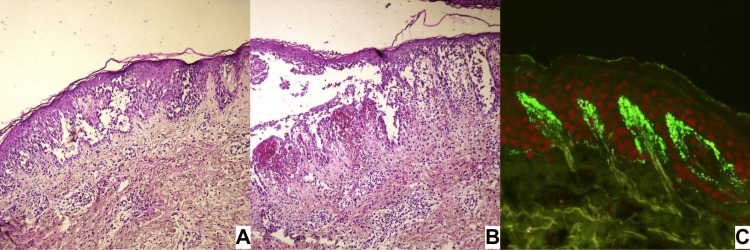
Histopathological and immunopathological findings. (A) Intraepidermal blister formation associated with inflammatory infiltrate (hematoxylin and eosin stain, original magnification ×100). (B) Higher magnification showing eosinophilic inflammatory infiltrate with scattered acantholytic cells (hematoxylin and eosin stain, original magnification ×200). (C) Direct immunofluorescence demonstrating granular IgA deposition predominantly at the tips of dermal papillae, confirming the diagnosis of dermatitis herpetiformis.

Skin biopsies were obtained from active lesions for histopathological examination, while perilesional skin was sampled for DIF analysis. Histopathological examination revealed intraepidermal blister formation associated with eosinophilic inflammatory infiltrate and scattered acantholytic cells (Figures 3A, 3B). Although these findings were atypical for DH and initially raised concern for pemphigus vulgaris and other autoimmune blistering disorders, clinicopathological correlation continued to favor an autoimmune blistering disorder despite the atypical microscopic findings.

DIF demonstrated granular IgA deposits predominantly localized at the tips of dermal papillae and focally along the basement membrane zone, establishing the diagnosis of DH (Figure 3C).

Initial management included oral cephalexin 500 mg four times daily for 10 days, potassium permanganate wet dressings, and strict gluten-free diet orientation. Baseline glucose-6-phosphate dehydrogenase levels were within normal limits. Following diagnostic confirmation, dapsone therapy was initiated at a dose of 100 mg/day, resulting in marked reduction of pruritus and substantial resolution of inflammatory lesions within two weeks. Dapsone was maintained for eight weeks without adverse effects. Progressive improvement in erosions, inflammatory activity, and overall skin condition was observed. At the six-month follow-up, the patient remained asymptomatic, without disease recurrence, while maintaining adherence to a strict gluten-free diet.

## Discussion

DH is a chronic IgA-mediated autoimmune blistering disease and represents the specific cutaneous manifestation of gluten-sensitive enteropathy [1]. Although classically characterized by grouped papulovesicular lesions on extensor surfaces, atypical and exuberant presentations may considerably hinder clinical recognition [2,6].

The present case is remarkable because of the coexistence of extensive erosive lesions, painful oral ulcerations, severe inflammatory activity, secondary bacterial infection, and atypical histopathological findings. These clinical and histopathological characteristics strongly favored alternative diagnoses, including Behçet disease, pemphigus vulgaris, and disseminated herpetic infection. 

Oral mucosal involvement is uncommon in DH and significantly increases diagnostic difficulty [7,8]. Indeed, oral lesions are rarely reported and, when present, are usually subtle and asymptomatic, although ulcerations, erythema, burning sensation, and vesiculobullous lesions have been described [8,9]. Furthermore, extensive excoriations and secondary infection may obscure the characteristic grouped papulovesicular morphology typically associated with DH.

Histopathologically, classic DH demonstrates subepidermal blister formation associated with neutrophilic microabscesses within dermal papillae [5]. Atypical histopathological findings may occur in manipulated, excoriated, or evolving lesions and can lead to diagnostic confusion with other autoimmune blistering diseases [10]. Furthermore, the absence of intercellular IgG deposition on DIF further argued against pemphigus vulgaris [11,12].

In the present case, positive anti-tissue transglutaminase and anti-endomysial antibodies provided additional evidence supporting the diagnosis and reinforced the underlying association between DH and gluten-sensitive enteropathy. Serological testing for celiac disease may be particularly useful in atypical presentations and should be considered as part of the diagnostic workup [1,3].

Although the patient did not report gastrointestinal symptoms and intestinal biopsy was not performed, the presence of positive anti-tissue transglutaminase and anti-endomysial antibodies, together with the characteristic DIF findings and excellent response to dapsone, strongly supported the diagnosis.

The rapid clinical response observed after initiation of dapsone further supported the diagnosis. Dapsone remains the first-line pharmacological treatment for DH and typically produces rapid symptomatic improvement, particularly regarding pruritus, often within days to weeks [3,13]. The patient experienced marked reduction in pruritus and inflammatory lesions within two weeks, with sustained remission during follow-up.

Several reports have highlighted the broad clinical spectrum of DH and emphasized the importance of immunopathological confirmation in unusual presentations [2,7,10,13]. Nevertheless, the present case differs from previously reported cases because of the combination of severe cutaneous involvement, oral lesions leading to suspicion of Behçet disease, hospitalization for presumed disseminated viral infection, atypical histopathological findings, and positive celiac serology.

The marked clinical improvement observed after initiation of dapsone and a gluten-free diet further reinforced the diagnosis and highlighted the therapeutic implications of establishing an early and accurate diagnosis.

## Conclusions

Exuberant DH with oral involvement may mimic several inflammatory, infectious, and autoimmune blistering disorders, including Behçet disease and disseminated herpetic infection, representing a significant diagnostic challenge.

This case emphasizes the importance of integrating clinical findings, histopathology, DIF, and celiac serology in atypical presentations of autoimmune blistering diseases. Early recognition of unusual manifestations of DH is essential to avoid delayed diagnosis, unnecessary hospitalizations, inappropriate therapies, and prolonged patient morbidity.
